# Sinus Floor Elevation From Palatal Approach With Simultaneous Implant Placement: A Case Report

**DOI:** 10.1155/crid/1108370

**Published:** 2026-07-07

**Authors:** Ding Jiamin, Guo Wen, Zhou Lin

**Affiliations:** ^1^ Department of Oral Mucosal Diseases, School and Hospital of Stomatology, Fujian Medical University, Fuzhou, Fujian, China, fjmu.edu.cn; ^2^ Institute of Stomatology Research Center of Dental and Craniofacial Implants, School and Hospital of Stomatology, Fujian Medical University, Fuzhou, Fujian, China, fjmu.edu.cn

**Keywords:** case report, implant placement, palatal approach, sinus floor elevation

## Abstract

**Introduction:**

In traditional maxillary sinus lift elevation with simultaneous implant placement with deficient residual bone in the posterior region of the maxilla, lateral window osteotomy is commonly employed. However, due to specific anatomical variations, such as an excessively thick buccal bone wall, a palatal approach with simultaneous implant placement may serve as a preferable alternative in selected cases.

**Case Presentation:**

A 48‐year‐old male requested implant‐supported restoration in the left maxillary posterior region. Preoperative cone‐beam computed tomography (CBCT) revealed insufficient vertical bone height with a residual bone height (RBH) of 2–3 mm. Given that the lateral wall of the maxillary sinus was significantly thicker than the palatal wall, whereas the alveolar ridge width was sufficient, a palatal approach was executed using the DASK kit for sinus osteotomy, accompanied by simultaneous implant placement. Postoperative CBCT confirmed a sinus graft height of 10 mm, with the bone graft material thoroughly surrounding the implants. Postoperative swelling was minimal. At the 7‐month postoperative follow‐up, CBCT demonstrated a continuous bone‐to‐implant interface without peri‐implant radiolucency, alongside a homogeneous radiopaque appearance of the grafted sinus blending with the native bone, confirming mature osteogenesis and permitting final prosthesis delivery.

**Conclusions:**

The palatal approach for maxillary sinus floor elevation with simultaneous implant placement is a technically feasible treatment modality under specific anatomical conditions.

## 1. Introduction

Following tooth extraction, the predictable resorption of alveolar bone combined with the progressive pneumatization of the maxillary sinus often results in a severely compromised amount of residual bone height (RBH) in the posterior maxilla. Maxillary sinus floor elevation (MSFE) is a widely adopted technique for augmenting bone volume in this region when managing cases characterized by insufficient RBH. Initially developed by Boyne and James in 1980 [1], the classical approach utilizes a lateral window technique. Subsequent advancements introduced simultaneous MSFE with implant placement, a modality that has demonstrated predictable long‐term clinical success with a survival rate exceeding 93% [2]. Conventionally, lateral window osteotomy is typically performed via a buccal approach due to its superior direct visibility and surgical convenience [3].

The maxillary sinus exhibits a highly intricate anatomical configuration, wherein the thicknesses of its lateral and palatal walls can vary significantly among individuals. Generally, the buccal wall is thinner than the palatal wall, featuring an average thickness of 1.95 ± 0.98 mm [4]. However, an anatomical variation exists.

Jadach et al. [5] reported that only 3% of patients present with a palatal wall that is thinner than the lateral wall. To accommodate these variations, fenestration for MSFE can be performed via buccal, palatal, or crestal osteotomies [6]. Although the buccal‐lateral approach remains the standard paradigm in implant dentistry [7], it is frequently associated with postoperative complications, including Schneiderian membrane perforation, substantial swelling, and hematoma [8], all of which may compromise patient comfort and satisfaction. Although a limited number of case reports have confirmed the feasibility of the palatal approach for MSFE, to the best of the authors′ knowledge, there is currently a paucity of literature documenting the palatal approach for MSFE performed in conjunction with simultaneous implant placement.

This case report is aimed at describing a novel palatal approach for MSFE with simultaneous implant placement in a patient presenting with a unique anatomical scenario, where the palatal bone wall was significantly thinner than the buccal side while maintaining a sufficient alveolar ridge width.

## 2. Case Presentation

A 48‐year‐old male patient presented to the Department of Implantology at the Affiliated Stomotological Hospital of Fujian Medical University with a chief complaint of missing teeth in the left posterior maxillary region. Clinical examination revealed the absence of Teeth #26 and #27, accompanied by third‐degree mobility of Tooth #24. The overlying gingival mucosa exhibited no pathological abnormalities, the interarch distance was within the normal limits, and a stable occlusal relationship was maintained.

**Figure 1:**
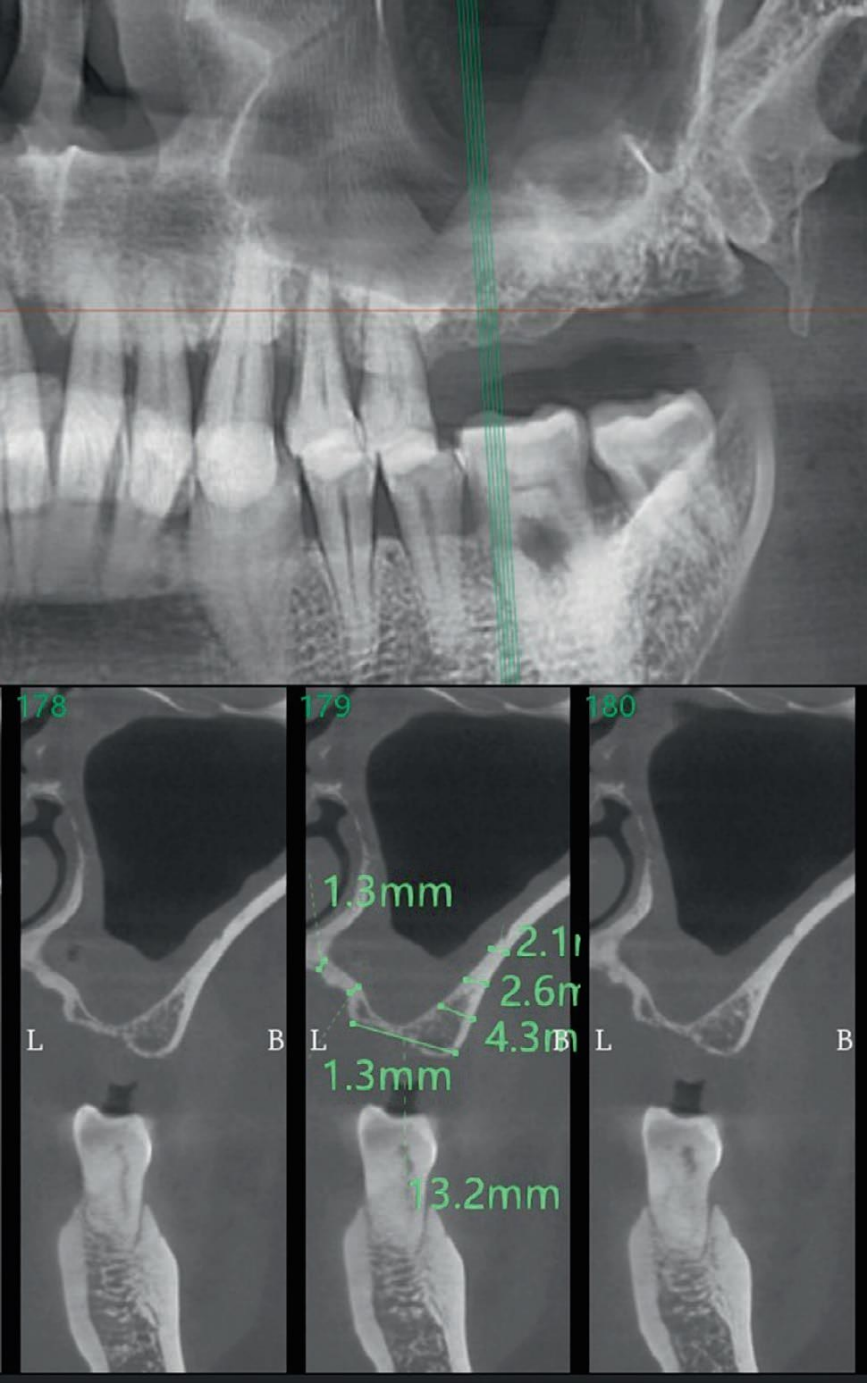
Preoperative CBCT scan of Tooth #26. The buccal sinus wall exhibits thicknesses of 4.3, 2.6, and 2.1 mm at the 3‐, 6‐, and 9‐mm levels, respectively. The palatal wall thicknesses at the 3‐ and 6‐mm levels are both 1.3 mm, and the width of the alveolar crest measures 13.2 mm.

**Figure 2:**
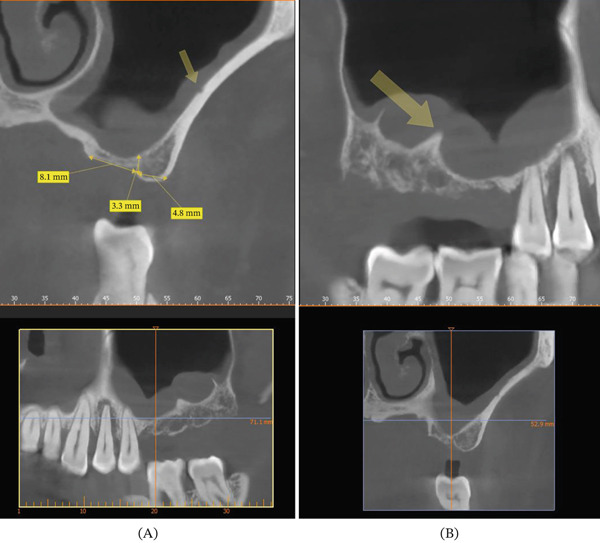
Preoperative CBCT demonstrated that there is a blood vessel in the bone wall of the lateral wall of (A) the maxillary sinus, and there is a septa formation between (B) Teeth #26 and #27.

Preoperative cone‐beam computed tomography (CBCT) revealed that the available vertical bone height in the posterior maxillary region was insufficient, with a RBH ranging between 2.0 and 3.0 mm, whereas the alveolar ridge width measured 11.7 mm. To establish a scientifically rigorous and reproducible anatomical baseline, the thicknesses of both the buccal and palatal sinus walls were quantitatively measured at three specific vertical levels directly above the lowest point of the maxillary sinus floor (3, 6, and 9 mm), in accordance with established radiographic protocols [9] the 3‐, 6‐, and 9‐mm levels, the buccal wall thicknesses were 4.3, 2.6, and 2.1 mm, respectively, confirming an unfavorable, excessively thick buccal plate. Conversely, the palatal wall thicknesses at the identical 3‐ and 6‐mm levels were 1.3 and 1.3 mm, respectively, demonstrating a significantly thinner cross‐sectional morphology (Figure 1). Furthermore, a prominent intraosseous blood vessel (alveolar antral artery) was identified traversing the buccal bone wall, and a bony septum was observed arising from the sinus floor between the roots of Sites #26 and #27 (Figure 2). Given the combined anatomical challenges—specifically, the high risk of membrane perforation during a blind transcrestal approach due to the septum, the hemorrhage risk associated with the buccal artery, and the favorable thinness of the palatal wall—a MSFE via a palatal approach with simultaneous implant placement was indicated. Prior to the sinus surgery, the compromised Premolar #24 was extracted due to severe periodontal disease.

The patient received local infiltration anesthesia with Primacaine (4% articaine, 1:100,000 adrenaline; ACTEON) extending from Site #24 to #27. Subsequently, full‐thickness midcrestal incisions were creatively outlined at Sites #24, #26, and #27, supplemented by an intrasulcus incision on the palatal aspect of Tooth #25 and a distal releasing incision to facilitate the reflection of an adequate palatal flap. During the elevation of the palatal mucoperiosteal flap, an unexpected arterial hemorrhagic event acutely occurred, secondary to an inadvertent laceration of a branch of the greater palatine artery (GPA). Profuse bleeding immediately obscured the surgical field. To ensure patient safety and restore visual clarity, ligation with 4‐0 resorbable sutures was performed.

Following successful stabilization of the surgical field, the localized treatment strategies were executed based on the site‐specific anatomy: An MSFE via a palatal approach was performed exclusively at Site #26, whereas standard ridge preparation was planned for Site #27. Osteotomy of the palatal window at the #26 region was performed using DASK drills (4# and 5#; XRT064025 and XTR084025, Dentium Implant System, Seoul, South Korea) to create a precisely demarcated round bone window. The Schneiderian membrane was initially detached from the sinus floor using a domed sinus curette (XSE1L, DASK, Dentium Implant System) and was subsequently elevated to the targeted height under direct visualization using a curved sinus curette (XSE3L and XSE4L, DASK, Dentium Implant System). Following this localized membrane elevation, standard implant site preparations were carried out along the crestal alveolar ridge at both Sites #26 and #27, with the osteotomy at Site #26 communicating with the elevated sinus cavity.

**Figure 3:**
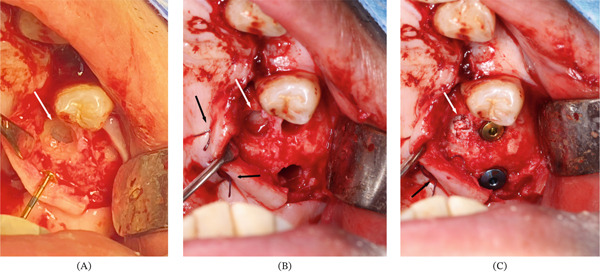
The brief process of the surgery. (A) The palatal flap elevation and a round bone window were created. (B) The maxillary sinus mucosa was lifted, and the implant site was prepared. (C) Xenogenic bone was filled in the sinus cavity, and the implant was placed. (White arrow: palatal maxillary sinus window; black arrow: suture knot for greater palatine artery ligation).

Xenogenic bone (Geistlich Bio‐Oss, large particles, 0.5 g) was incrementally packed into the newly created subantral space at Site #26 through both the palatal bone window and the corresponding crest implant osteotomy, with gentle pressure directed toward the buccal aspect to optimize distribution. Thereafter, a 4.2 × 8 − mm and 4.8 × 6 − mm implants (Astra EV, Astra Tech Implant System, Dentsply Sirona, Charlotte, United States) were sequentially placed at Sites #26 and #27, respectively (Figure 3). Specifically, the implant at Site #26 was stabilized within the augmented sinus site, whereas the implant at Site #27 was anchored entirely within the native ridge bone. Additional xenogenic bone graft was introduced via the palatal window to thoroughly adapt around the exposed implant fixtures within the #26 sinus cavity and to secure adequate subantral bone volume. A resorbable collagen membrane was then applied to cover the palatal bone window, after which the mucoperiosteal flap was repositioned and primary closure was achieved with interrupted sutures. The patient underwent CBCT examination immediately after surgery. To minimize postoperative sequelae and prevent infection, the patient was prescribed systemic antibiotics (cefaclor, 250 mg tid; Tinidazole, 500 mg bid) for 5 days, alongside a 0.12% chlorhexidine mouthrinse for 1 week. Sutures were removed 2 weeks postoperatively, showing uneventful wound healing.

**Figure 4:**
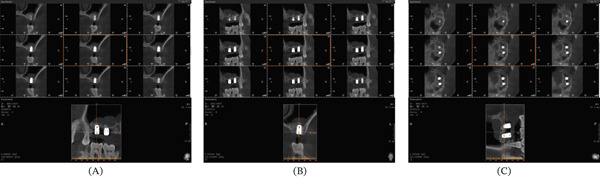
Postoperative CBCT demonstrated that the bone graft materials were adequately wrapped around the implant roots. (A) Sagittal cross‐section. (B) Coronal cross‐section. (C) Axial cross‐section.

Postoperative CBCT confirmed that the Schneiderian membrane had been successfully elevated by 10 mm from the alveolar crest. The three‐dimensional positioning of the implants was acceptable, and the graft materials provided circumferential coverage around the implant bodies (Figure 4).

**Figure 5:**
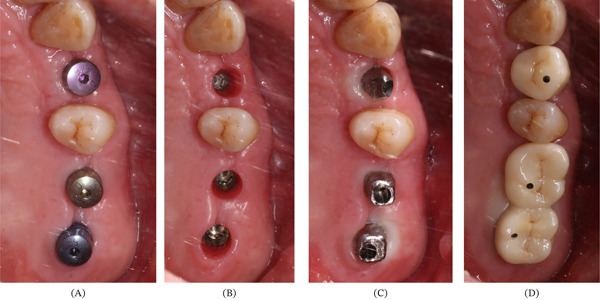
The restoration of Molars #26 and #27. (A) Healing abutments. (B) Transmucosal cuff. (C) Personalized base abutments. (D) Zirconia ceramic crowns.

**Figure 6:**
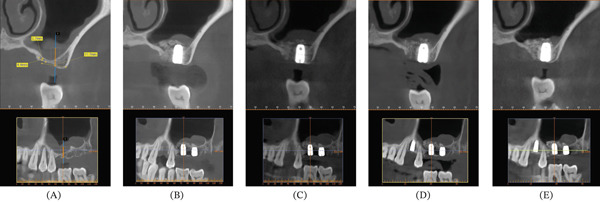
The preoperative design, postoperative, and follow‐up CBCT. (A) Preoperative design. (B) Postoperative CBCT. (C) One month postoperative CBCT. (D) Two months postoperative CBCT. (E) Seven months postoperative CBCT.

At the 1‐month follow‐up, clinical examination confirmed stable healing at the extraction socket of Site #24, and CBCT revealed no signs of sinus pathology or graft migration around the molar implant site (#26). Two months postsurgery, an implant was successfully placed at the Premolar #24 site; follow‐up CBCT reconfirmed stable graft volume and uneventful healing in the left maxillary sinus. Seven months postoperatively, the patient returned for definitive prosthetic rehabilitation. The follow‐up CBCT scans demonstrated continuous bone‐to‐implant contact without any peri‐implant radiolucency, confirming successful osseointegration and excellent graft consolidation. Premolar #24 was restored with a single zirconia ceramic crown (Zenostar, Wieland Dental + Technik), whereas Molars #26 and #27 were rehabilitated using splinted zirconia crowns supported by customized titanium abutments (Figure 5). The comprehensive treatment workflow, including the preoperative planning, immediate postoperative outcomes, and long‐term follow‐up CBCT scans, is illustrated in Figure 6.

## 3. Discussion

This case report demonstrates the clinical feasibility and predictability of executing a site‐specific MSFE via a palatal approach exclusively at Site #26, combined with simultaneous implant placement.

When managing cases with a severely compromised RBH of 2.0–3.0 mm, the standard clinical consensus predominantly dictates the utilization of a traditional buccal‐lateral window technique for MSFE to ensure adequate graft volume and primary implant stability. However, a conventional buccal‐lateral approach was highly precluded in our present case due to an exceptionally thick buccal bone wall (up to 4.2 mm) combined with a prominent intraosseous alveolar antral artery traversing the precise path of the planned osteotomy, making an approach from the buccal side highly hazardous. Alternatively, a minority of investigators have reported the feasibility of managing a 2.0–3.0 mm RBH via a transcrestal internal elevation under strict protocols [10]. Nevertheless, such a transcrestal approach was highly unsuitable for this patient due to the presence of a sharp antral septum between Sites #26 and #27. Implementing a transcrestal lift under these anatomical conditions relies entirely on blind tactile maneuvering, which entails exceptionally high technique sensitivity. Because the Schneiderian membrane exhibits extremely limited elasticity when stretched over sharp bony ridges—as established by the biomechanical principles of Pommer et al. [11]—attempting a blind 4.0–5.0‐mm tactile elevation in an environment with only 2.0–3.0 mm of RBH intersected by a septum carries an unacceptably high risk of membrane perforation. Consequently, with both the standard buccal‐lateral window and the blind transcrestal internal lift ruled out due to these prohibitive anatomical constraints, exploiting the remarkably thin palatal wall (0.9–1.2 mm) emerged as the ideal, mandatory strategy, providing a low‐trauma cortical window for direct‐vision membrane detachment exclusively at Site #26.

Patient‐centered outcomes in this report presented a valuable disparity between postoperative swelling and pain. In consensus with Moreno Rodríguez. et al. [12], facial swelling was remarkably minimal. This is primarily because avoiding buccal flap reflection preserves the supraperiosteal vascular plexus of the cheek, preventing visible fluid accumulation. Regarding postoperative pain, although a standardized quantitative pain scale or precise analgesic pill tracking was not implemented due to the retrospective nature of this case, the patient′s subjective experience was thoroughly evaluated during the scheduled 2‐week suture removal appointment. Upon retrospective inquiry regarding the entire healing phase, the patient provided highly favorable feedback, explicitly stating that the surgical site experienced no distinct facial swelling and was “not particularly painful,” characterizing the immediate postoperative days as involving only mild, easily tolerable discomfort.

Despite these advantages, the palatal approach presents inherent technical ergonomics limitations and vital vascular risks [13]. The GPA represents a critical anatomical hazard during palatal flap reflection; accidental laceration can cause profuse hemorrhage that severely obscures the surgical field. In our case, this complication was proactively prevented through anatomical‐guided flap design and precise ligation at the flap base.

The localized palatal approach for MSFE with simultaneous implant placement serves as a powerful, technique‐sensitive alternative to the standard buccal approach. It is strictly indicated under precise criteria: an unfavorable buccal‐to‐palatal thickness gradient, prominent buccal intraosseous vessels, and localized septa obstacles, provided there is a sufficient transversal ridge width to accommodate palatal instrumentation [14]. Crucially, the predictability of executing simultaneous implant placement in an environment with a severely limited RBH (2.0–3.0 mm) hinges entirely on achieving sufficient initial primary stability. The synchronous single‐stage implant placement significantly reduces the overall rehabilitation period and patient morbidity, provided that the regional bone architecture can guarantee mechanical interlocking [15]. In our case, although the vertical bone height was profoundly compromised, the patient exhibited an optimal transversal ridge width. This residual three‐dimensional bone volume offered sufficient cortical anchorage to frictionally engage the implant fixtures during insertion.

## 4. Conclusion

The palatal approach for MSFE with simultaneous implant placement is a technically feasible treatment modality under specific anatomical conditions.

## Author Contributions

Ding Jiamin: data curation and writing—original draft. Guo Wen: investigation and supervision. Zhou Lin: writing—review and editing and funding acquisition.

## Funding

This study was supported by the National Natural Science Foundation of China (10.13039/501100001809) (82571054) and Fujian Provincial Health Technology Project (2024GGA068).

## Disclosure

All authors have read and approved the final version of the manuscript. Zhou Lin had full access to all of the data in this study and takes complete responsibility for the integrity of the data and the accuracy of the data analysis. A preprint has previously been published (Jiamin et al. [16]).

## Ethics Statement

Institutional review board approval was waived for this single anonymized case report. Written informed consent was obtained from the patient for the publication of this case report and any accompanying images.

## Conflicts of Interest

The authors declare no conflicts of interest.

## Data Availability

The data that support the findings of this study are available from the corresponding author upon reasonable request.
